# Rational Design of a Small Molecular Near-Infrared Fluorophore for Improved In Vivo Fluorescence Imaging

**DOI:** 10.3390/ma16227227

**Published:** 2023-11-18

**Authors:** Gayoung Jo, Yoonbin Park, Min Ho Park, Hoon Hyun

**Affiliations:** 1 Department of Biomedical Sciences, Chonnam National University Medical School, Hwasun 58128, Republic of Korea; 2 BioMedical Sciences Graduate Program (BMSGP), Chonnam National University, Hwasun 58128, Republic of Korea; 3 Department of Surgery, Chonnam National University Medical School and Hwasun Hospital, Hwasun 58128, Republic of Korea

**Keywords:** small molecular fluorophores, near-infrared fluorophores, near-infrared fluorescence imaging, targeted imaging, tumor targeting

## Abstract

The near-infrared (NIR) fluorescence imaging modality has great potential for application in biomedical imaging research owing to its unique characteristics, such as low tissue autofluorescence and noninvasive visualization with high spatial resolution. Although a variety of NIR fluorophores are continuously reported, the commercially available NIR fluorophores are still limited, owing to complex synthetic processes and poor physicochemical properties. To address this issue, a small molecular NIR fluorophore (SMF800) was designed and developed in the present work to improve in vivo target-specific fluorescence imaging. After conjugation with pamidronate (PAM) and bovine serum albumin (BSA), the SMF800 conjugates exhibited successful in vivo targeting in bone and tumor tissues with low background uptake, respectively. The improved in vivo performance of the SMF800 conjugate demonstrated that the small molecular NIR fluorophore SMF800 can be widely used in a much broader range of imaging applications. The structure of SMF800, which was developed by considering two important physicochemical properties, water solubility and conjugatability, is first introduced. Therefore, this work suggests a simple and rational approach to design small, hydrophilic, and conjugatable NIR fluorophores for targeted bioimaging.

## 1. Introduction

The near-infrared (NIR) fluorescence imaging system is an important modality for basic research, preclinical, and clinical applications because of its advantageous features, such as low cost, portability, low tissue autofluorescence, and noninvasive visualization with high spatial resolution in real-time [1,2,3]. Since the optical imaging modality in the NIR window (650–950 nm) can overcome several limitations of traditional medical imaging modalities, such as computed tomography (CT), positron emission computed tomography (PET), single photon emission computed tomography (SPECT), and magnetic resonance imaging (MRI), target-specific NIR fluorophores are necessarily important and play a crucial role in optimizing clinical benefits of the NIR fluorescence imaging technique [4,5,6]. However, there are only a few NIR fluorophores available clinically, of which the U.S. Food and Drug Administration (FDA)-approved indocyanine green (ICG) predominates. Although ICG is extensively used for cardiac/hepatic function tests, lymph node localization, ureteral detection, and chorioretinal fluorescence angiography, ICG is far from ideal because of its low water solubility, stability, and high protein binding, as well as a lack of chemically conjugatable groups [7,8,9,10,11]. Hence, commercially available NIR fluorophores have been produced and typically utilized for in vitro and in vivo NIR fluorescence imaging after combining with various small-molecule drugs, peptides, proteins, and polymers [12,13,14,15].

Despite great efforts to develop many types of NIR fluorophores, the target specificity of their conjugates could be changed depending on the physicochemical properties of the conjugated NIR fluorophore, mainly hydrophobicity, polarity, and net surface charge [16,17,18]. Among the commercial NIR fluorophores, anionic IRDye800CW and Cy5.5 showed high nonspecific tissue/organ uptake in vivo, both before and after conjugation with targeting ligands, because of their highly anionic surface charges [19]. One common feature of the commercially available NIR fluorophores is that the negatively charged sulfonate groups are required to increase hydrophilicity and water stability. Previously, a hydrophobic cationic NIR fluorophore, named IR-786, was used for mitochondria-targeted imaging with high fluorescence intensity [20]. In addition, Choi et al. reported in vitro anticancer activity of IR-786 conjugated with anticancer drugs, which displayed enhanced anticancer effects on patient-derived glioblastoma cells [21,22]. Nevertheless, the high hydrophobicity and biological toxicity of IR-786 limit its application in bioimaging. To solve this problem, ZW800-1, known as a hydrophilic and zwitterionic NIR fluorophore, was first introduced by Choi et al. It displayed outstanding in vivo performance due to low nonspecific uptake in organs/tissues and rapid renal elimination from the body within a few hours, thereby providing high target-to-background ratios after combining with diverse targeting ligands [17,19,23,24]. Taken together, the most important considerations to design the conjugatable NIR fluorophores for improving in vivo performance are water solubility and net surface charge of the cyanine-based NIR fluorophores.

Typically, the physicochemical characteristics of polymethine-based NIR fluorophores rely on their chemical and geometric configuration, which is significantly important to determine the in vivo biodistributions in terms of blood half-life, body clearance, and targeting efficiency [19]. That is, an increase in water solubility could enhance optical properties and improve in vivo biodistribution accomplished by altering the functional groups of the cyanine-based NIR fluorophores. Additionally, their large molecular weights of more than 1000 Da could disrupt the retention of bioactivity after conjugating with small-molecule drugs and peptides [25]. In this regard, the chemical structures of conventional cyanine-based NIR fluorophores should be modified to reduce their molecular weights to less than 500 Da, while maintaining the surface charge balance in the entire structure. Although other types of seminaphthofluorones and azaphosphinate dyes were also developed as low molecular weight and water-soluble fluorophores, their structures are based on the phenoxazine backbone, which involves complicated synthesis.

**Scheme 1:**
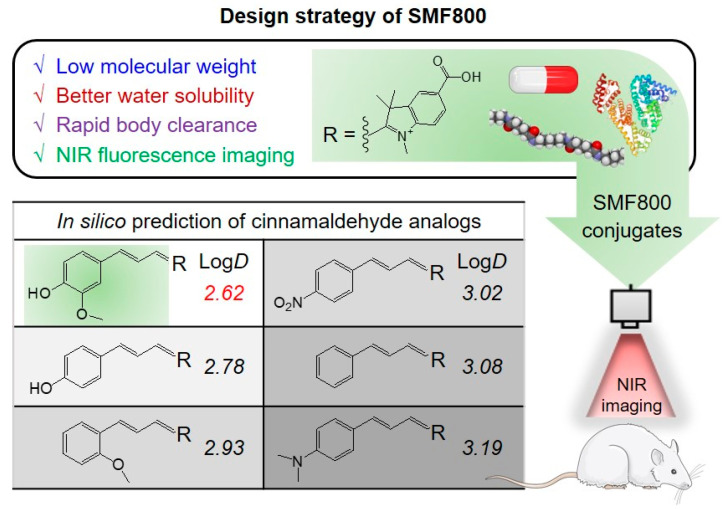
Rational design strategy of cinnamaldehyde-based small molecular NIR fluorophores for conjugation with various drugs, polymers, and proteins. The 4-hydroxy-3-methoxy cinnamaldehyde was finally selected from cinnamaldehyde analogs after in silico calculations of their hydrophobicity (log*D* at pH 7.4) values.

Herein, we report a new type of small molecular NIR fluorophore, named SMF800, which shows fast renal and hepatobiliary excretion from the body to improve the target-specific NIR fluorescence imaging (Scheme 1). The SMF800 NIR fluorophore armed with a conjugatable carboxyl group was prepared by a simple synthetic method for the NIRemitting cyanine-based structure. This is the first report of the chemical structure of SMF800 and the design of a small molecular NIR fluorophore consisting of a carboxylated indole moiety and a hydrophilic cinnamaldehyde moiety. One of the important advantages of this approach is the versatility of SMF800, making it possible to change the conjugatable groups with amine or cleavable linkers according to their counterpart molecules and to control the hydrophobicity by modifying the backbone structures. Thus, SMF800 is comparable with conventional polymethine cyanine dyes in terms of molecular weight, water solubility, and in vivo biodistribution. In a proof of concept, the SMF800 NIR fluorophore was conjugated with small-molecule drugs and proteins, respectively, to confirm the biodistribution and clearance behavior of SMF800 before and after conjugation. Consequently, two different types of SMF800 conjugates developed in the present study revealed target-specific in vivo performance with low nonspecific background uptake. Therefore, this study demonstrates the potential utility of the small molecular NIR fluorophore SMF800 by conjugation with various drugs and biomolecules for targeted NIR fluorescence imaging.

## 2. Materials and Methods

### 2.1. Synthesis of Small Molecular NIR Fluorophore (SMF800)

All chemicals used in the present work were received from Sigma-Aldrich (St. Louis, MO, USA). 4-hydrazinobenzoic acid **1** (1 g, 6.6 mmol) and 3-methyl-2-butanone **2** (1.1 mL, 9.9 mmol) were placed into glacial acetic acid (15 mL). The reaction mixture was refluxed for 8 h, and the reaction solvent was eliminated using a rotavapor. A solution of water and methanol (10 mL, 90/10 *v*/*v*%) was used to dissolve the reaction residue. The undissolved product was filtered off, the filtrate was stored at ambient temperature, and the yellow crystal **3** (0.9 g, 67%) was obtained by filtration. A mixture of carboxylated indole **3** (0.5 g, 2.5 mmol) and iodomethane (1.22 mL, 3.8 mmol) suspended in acetonitrile (15 mL) was refluxed for 18 h. The precipitate intermediate **4** was obtained by filtration and washed with acetonitrile and ethyl acetate. The obtained product was used for the next reaction step without further purification (0.6 g, 70%). A mixture of heterocyclic salt **4** (0.1 g, 0.29 mmol), 4-hydroxy-3-methoxy cinnamaldehyde **5** (0.046 g, 0.26 mmol), and anhydrous sodium acetate (0.04 g, 0.48 mmol) dissolved in absolute ethanol (5 mL) was refluxed for 6 h. The reaction mixture was cooled and then dried by a rotavapor to remove the reaction solvent, and collected as dark-red powder (SMF800; 0.08 g, 81%). The crude reaction mixture was loaded onto a preparative high-performance liquid chromatography (HPLC) system (Waters, Milford, MA, USA) to separate the target product. Finally, the accurate mass of the purified SMF800 was analyzed by the Dionex UltiMate^TM^ 3000 mass spectrometry system (Thermo Scientific, Waltham, MA, USA).

### 2.2. Optical and Physicochemical Property Analyses

Absorbance and fluorescence analyses of SMF800 were performed using phosphate-buffered saline (PBS) at pH 7.4. The absorption spectrum of SMF800 was analyzed by a fiber optic UV-Vis-NIR (200–1025 nm) spectrometer (Ocean Optics, Dunedin, FL, USA). The Beer-Lambert equation was used to calculate the molar extinction coefficient (*ε*). To measure the fluorescence quantum yield (*Φ*) of SMF800, ICG (*Φ* = 13% measured in dimethyl sulfoxide) was used as a calibration standard by matching the absorbance at 770 nm [26,27]. The fluorescence emission spectrum of SMF800 was recorded using a SPARK^®^ 10M microplate reader (Tecan, Männedorf, Switzerland) at an excitation wavelength of 750 nm and emission wavelengths ranging from 770 to 900 nm. In silico predictions of the log*D* at pH 7.4 and topological polar surface area (TPSA) were performed using Marvin and JChem calculator plugins (ChemAxon, Budapest, Hungary).

### 2.3. Preparation of SMF800 Conjugates

Pamidronate (PAM) and bovine serum albumin (BSA) were received from Sigma-Aldrich and used without further purification. For SMF800-PAM, SMF800 (1 mg, 2.6 μmol) was conjugated to PAM (0.6 mg, 2.6 μmol) in distilled water (DW; 1 mL) by mixing with 4-(4,6-dimethoxy-1,3,5-triazin-2-yl)-4-methylmorpholinium chloride (DMT-MM; 1 mg, 3.6 μmol) at room temperature for 12 h. To prepare the BSA-SMF800 conjugate, SMF800 (0.1 mg, 0.26 μmol) was conjugated to BSA (17 mg, 0.26 μmol) by adding DMT mm (0.18 mg, 0.65 μmol) into DW (1 mL) at room temperature for 24 h. The reaction mixture was purified through a gel-filtration chromatography (GFC) system attached to Econo-Pac P6 cartridges (Bio-Rad, Hercules, CA, USA) at a fixed flow rate (1 mL/min using 0.1X PBS, pH 7.4). The hydrodynamic diameter of BSA-SMF800 conjugate was monitored at 280 nm through an ÄKTA start™ system (GE Healthcare, Piscataway, NJ, USA).

### 2.4. NCI-H460 Xenograft Mouse Model

Animal experiments were carried out in accordance with the guidelines approved by the Chonnam National University Animal Research Committee (CNU IACUC-H-2022-43). Male athymic nude mice (6 weeks old and ≈25 g, *n* = 3 independent experiments) were purchased from OrientBio (Gwangju, Republic of Korea) to prepare human tumor xenograft models. The human large-cell lung carcinoma cell line (NCI-H460) was purchased from the American Type Culture Collection (ATCC, Manassas, VA, USA). NCI-H460 was cultured in Roswell Park Memorial Institute (RPMI) 1640 medium (Gibco BRL, Paisley, UK) containing fetal bovine serum, penicillin, streptomycin, and amphotericin B (Welgene, Daegu, Republic of Korea) on a culture plate. The cultured cells were stored in a humidified incubator set to 5% CO_2_ at 37 °C. The cultured cancer cells were dispersed in PBS before inoculation subcutaneously into the mouse upper right flank area (1 × 10^6^ cells per mouse). Finally, mice bearing subcutaneous tumors sized with an average diameter of 1 cm were subjected to intravenous injection of SMF800 and its conjugates at 8–10 days post-inoculation. The tumor-bearing mice were anesthetized at specific time points for real-time whole-body imaging.

### 2.5. In Vivo NIR Fluorescence Imaging

In vivo NIR fluorescence imaging was conducted using a FOBI imaging system (CellGenTek, Deajeon, Republic of Korea). Tumor-bearing mice treated with each fluorescent sample (*n* = 3 in each experiment group) were, respectively, imaged and sacrificed at 1, 4, or 24 h after the intravenous injection. Tumor tissues and major organs (including heart, lungs, liver, pancreas, spleen, kidneys, duodenum, and intestine) were collected at specific time points to perform quantitative fluorescence analysis of each sample. The values of fluorescence intensities were obtained using the ImageJ program (National Institutes of Health, Bethesda, MD, USA).

## 3. Results

### 3.1. Synthesis and Characterization of SMF800

**Figure 1:**
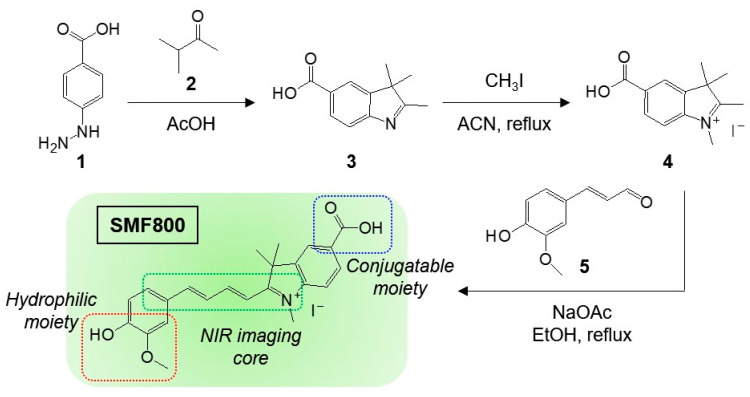
Synthetic scheme of cinnamaldehyde-based small molecular NIR fluorophore SMF800.

The cinnamaldehyde-based small molecular NIR fluorophore, SMF800, was designed for labeling biomolecules and prepared as shown in Figure 1. With an aim to improve the hydrophilicity of SMF800, the 4-hydroxy-3-methoxy cinnamaldehyde was optimally selected among the commercially available other cinnamaldehyde analogs after in silico calculations of their distribution coefficient (log*D*) values (2.62–3.19), as shown in Scheme 1. The small molecular NIR fluorophore SMF800 was readily prepared by the condensation reaction between carboxylated indolium (**4**) and the chosen 4-hydroxy-3-methoxy cinnamaldehyde (**5**) in the presence of anhydrous sodium acetate. The SMF800 structure consists of three important parts, which are the carboxylated indole moiety for covalent conjugation with amine groups, the methine bridges for NIR fluorescence imaging, and the hydroxy and methoxy-substituted benzene ring for the improvement of hydrophilicity. The carboxylated indole may also be easily modified according to the counterpart molecules to be conjugated. Additionally, other types of cinnamaldehyde derivatives can be used to adjust the hydrophobicity and excitation/emission wavelength. This synthetic procedure provides a simple and efficient approach to design small, hydrophilic, and conjugatable NIR fluorophores for labeling biomolecules.

**Figure 2:**
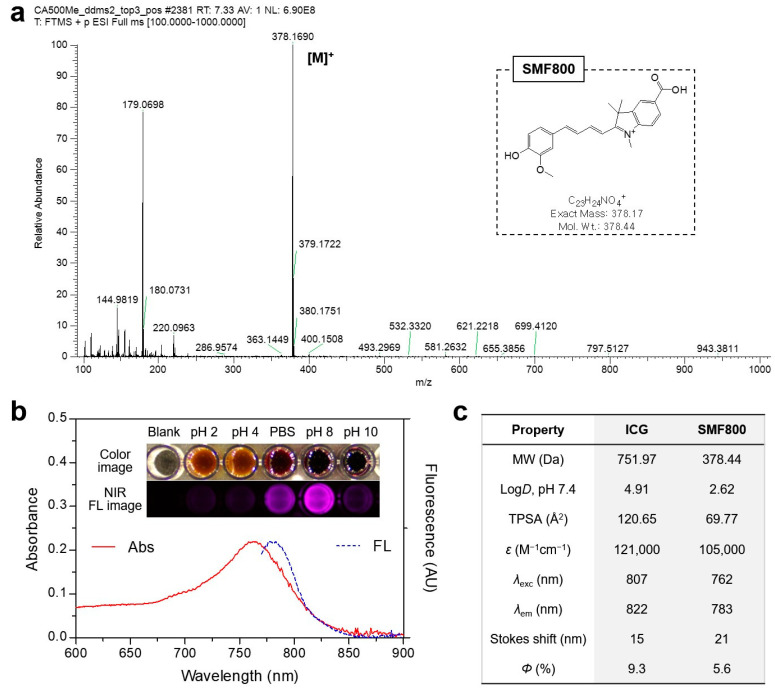
(**a**) Mass spectrum of the purified SMF800 NIR fluorophore. (**b**) Absorbance and fluorescence emission spectra of SMF800 observed in PBS at pH 7.4. The inset shows the fluorescence image of SMF800 by changing pH conditions. (**c**) Physicochemical and optical properties of ICG [28] and SMF800. In silico calculations of log*D* and TPSA were performed using the Marvin and JChem calculator plugins (ChemAxon).

Subsequently, the accurate mass of SMF800 was successfully analyzed by time-of-flight mass spectrometry, after separation of the reaction mixture (Figure 2a). The exact mass (i.e., the theoretical mass; *m*/*z* 378.17) of SMF800 is the calculated mass of an ion with this elemental formula, isotopic composition, and charge state. The accurate mass (i.e., the experimental mass; *m*/*z* 378.1690) of SMF800 is the determined mass of an ion measured to an appropriate degree of accuracy and precision used to determine the successful synthesis of SMF800. Also, the optical properties of the purified SMF800 were determined in PBS at pH 7.4. The wavelengths of maximum absorption and emission of SMF800 showed at 762 and 783 nm in the NIR window, respectively, with 21 nm Stokes shift (Figure 2b). This demonstrates that the NIR-emitting SMF800 fluorophore can be used for NIR fluorescence imaging after conjugating with various biomolecules. Interestingly, the fluorescence intensity of the SMF800 NIR fluorophore does depend on the pH conditions and decreases significantly in an acidic environment. At pH 8, the SMF800 exhibited stronger fluorescence intensity than that of neutral pH conditions. At the high alkaline pH range (pH 10), the fluorescence intensity of SMF800 was considerably reduced. Thus, the cinnamaldehyde-based SMF800 NIR fluorophore may have an advantage for use as fluorescent indicators for monitoring alkaline pH changes. As summarized in Figure 2c, in silico prediction of the physicochemical characteristics, such as hydrophilicity and chemical polarity, was performed using ChemAxon’s JChem software (under version 14.12.15.0). SMF800 displayed a relatively higher hydrophilicity (log*D* = 2.62) and polarity (TPSA = 69.77 Å^2^), as compared with in silico predictions of other structures shown in Scheme 1. Also, SMF800 exhibited a moderate molar extinction coefficient (*ε* = 105,000 M^−1^ cm^−1^) and quantum yield (*Φ* = 5.6%) compared to that of the commercially available and clinically approved contrast agent ICG. Taken together, the improved physicochemical properties of SMF800 may not only contribute to better optical properties but also to the best in vivo performance.

### 3.2. Time-Dependent In Vivo Biodistribution of SMF800

**Figure 3:**
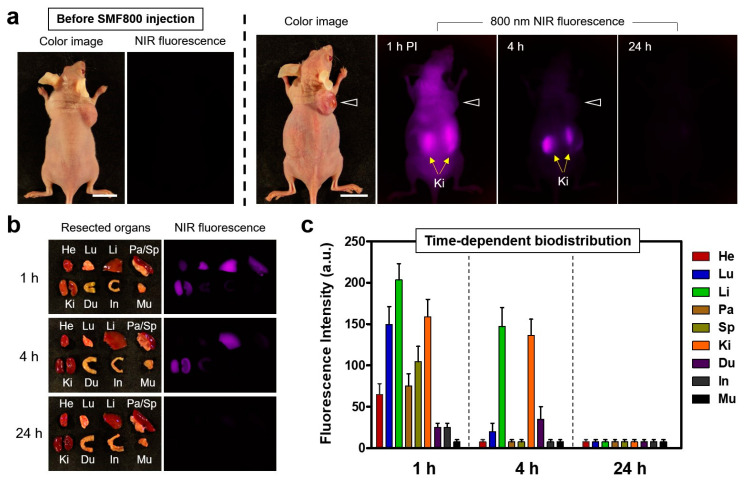
Time-dependent in vivo biodistribution of SMF800. (**a**) Real-time NIR fluorescence imaging 24 h after SMF800 injection. Arrowheads indicate the tumor sites. (**b**) Biodistributions observed by major organs harvested at 1, 4, and 24 h after SMF800 injection. Abbreviations: Du, duodenum; He, heart; In, intestines; Ki, kidneys; Li, liver; Lu, lungs; Mu, muscle; Pa, pancreas; Sp, spleen; PI, post-injection. Scale bars = 1 cm. (**c**) Quantitative fluorescence analysis of intraoperative dissected organs 24 h after injection of SMF800.

Time-dependent in vivo biodistribution and tumor accumulation of SMF800 were observed in an NCI-H460 xenograft mouse model. Twenty nmol of SMF800 (200 µM concentration in 100 µL PBS) was injected intravenously into the tumor-bearing mice, then the mice were monitored for 24 h through an NIR fluorescence imaging system in real-time (Figure 3a). Time-dependent NIR fluorescence monitoring showed no significant accumulation of SMF800 in tumor tissues until 24 h after injection. More importantly, the fluorescence signals in the whole body were rapidly decreased and detected only in the kidneys in the prone position within 4 h post-injection. Finally, no fluorescence signals in the body were observed at 24 h after injection of SMF800. This demonstrates that the small and zwitterionic structure of SMF800 may play a key role in rapid clearance within 4 h of injection, thereby facilitating the improved target-specific imaging after conjugation with various biomolecules. Additionally, we reconfirmed the biodistribution of the SMF800 in major organs harvested from the mice (Figure 3b,c). The SMF800 mainly accumulated in the liver and kidneys due to renal and hepatic clearance within 4 h post-injection and was completely eliminated from the body at 24 h post-injection. Therefore, the SMF800 can be optimally used as a labeling agent without nonspecific background uptake, which is one of the major challenges facing conventional NIR fluorophores including commercially available IRDye800CW and Cy5.5.

### 3.3. Time-Dependent In Vivo Bone Imaging of SMF800-PAM Conjugate

**Figure 4:**
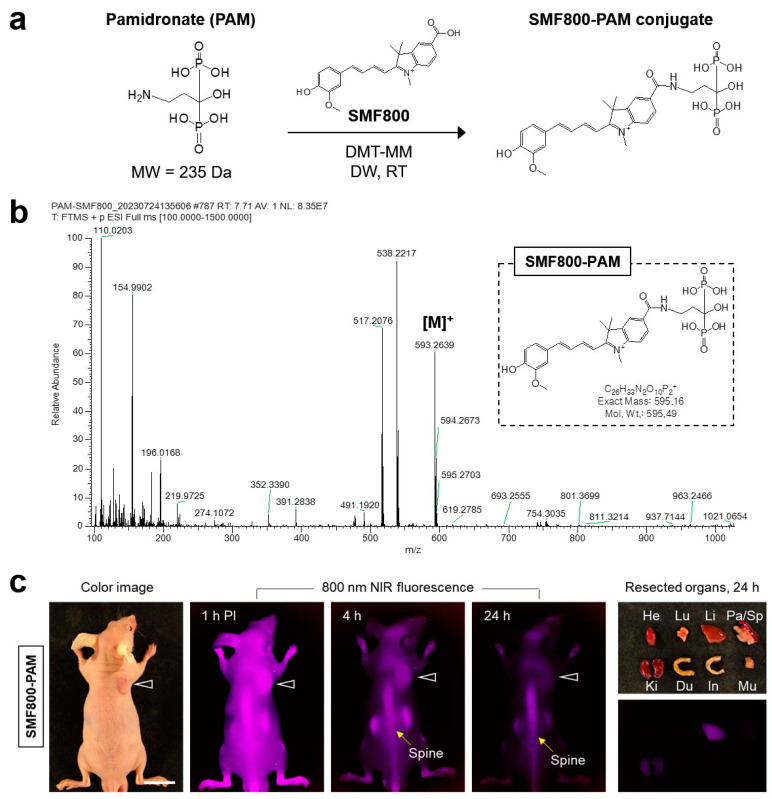
Time-dependent in vivo bone targeting efficiency of the SMF800-PAM conjugate. (**a**) Scheme of conjugation reaction between SMF800 and PAM. (**b**) Mass spectrum of the SMF800-PAM conjugate. (**c**) In vivo and ex vivo NIR fluorescence imaging (whole body and resected major organs) 24 h after injection of the SMF800-PAM conjugate. Arrowheads indicate the tumor sites. Scale bars = 1 cm.

PAM, as a small-molecule drug, was used for the first example of the SMF800 conjugation. Previously, bone-specific NIR fluorophores were developed by conjugating the commercial NIR fluorophores such as IRDye78, IRDye800CW, and ZW800-1 with PAM [29]. However, the bone specificity of PAM was highly affected by the physicochemical properties of the conjugated NIR fluorophores, especially due to the net surface charges. To improve the bone-specific imaging of PAM, the carboxyl group of SMF800 was covalently conjugated with the amine group of PAM, facilitated by a water-soluble DMT-MM coupling agent (Figure 4a). The DMT-MM plays an important role in the activation of the carboxyl group of SMF800; then, the amide bond can be formed between the electrophilic carboxylic acid ester of SMF800 and the amine group of PAM by a nucleophilic substitution reaction. After the conjugation reaction, the molecular weight of SMF800-PAM was successfully confirmed by mass spectrometry (Figure 4b). After confirming the successful conjugation between PAM and SMF800, 20 nmol of SMF800-PAM conjugate (200 µM concentration based on SMF800 in 100 µL PBS) was administered intravenously into tumor-bearing mice and the mice were monitored for 24 h (Figure 4c). Interestingly, the tumors showed no significant uptake of SMF800-PAM until 24 h post-injection. As expected, the high fluorescence signals in the bone tissues, especially spine, were prominently displayed at 24 h post-injection, because the background fluorescence signals in the body gradually decreased for 24 h after injection. This result demonstrates that the SMF800 was successfully used for in vivo bone-specific imaging after conjugation with PAM.

### 3.4. In Vivo Tumor Targeting of BSA-SMF800 Conjugate

**Figure 5:**
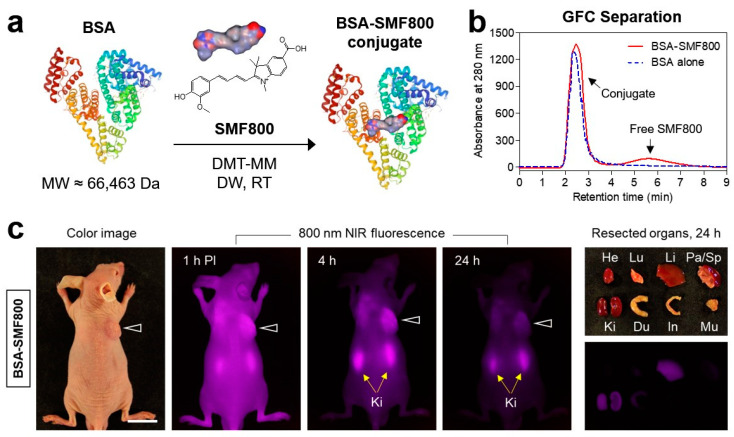
Time-dependent in vivo tumor targeting efficiency of the BSA-SMF800 conjugate. (**a**) Scheme of conjugation reaction between BSA and SMF800. (**b**) GFC separation of BSA alone and BSA-SMF800 conjugate. (**c**) In vivo and ex vivo NIR fluorescence imaging (whole body and resected major organs) 24 h after injection of the BSA-SMF800 conjugate. Arrowheads indicate the tumor sites. Scale bars = 1 cm.

Finally, BSA was employed as a tumor-targeted protein for an additional example of SMF800 conjugation. BSA is a well-known protein utilized as multi-purpose carriers, owing to its biocompatibility, biodegradability, non-immunogenicity, cost-effectiveness, and high load-carrying capacity. According to the enhanced permeability and retention (EPR) effect, BSA can be used for targeted tumor accumulation and efficient drug delivery [30]. Moreover, the selective tumor accumulation of BSA can also be demonstrated by receptor-mediated albumin uptake pathways involved with membrane-associated albumin-binding proteins, including secreted protein acidic and rich in cysteine (SPARC) [30]. Previously, our group reported that BSA could be utilized as a fluorescent probe carrier for extended blood circulation and improved tumor accumulation of commercial NIR fluorophores including ICG and IR-786 [28]. To use the BSA macromolecules for tumor-targeted imaging, SMF800 was covalently conjugated with the amine groups of BSA in the presence of DMT-MM for the formation of amide bonds (Figure 5a). Subsequently, the reaction mixture was separated by a GFC system to remove the unreacted SMF800 and confirm the retention times between BSA alone and BSA-SMF800 conjugate (Figure 5b). After purifying the BSA-SMF800 conjugate, 20 nmol of BSA-SMF800 conjugate (200 µM concentration based on SMF800 in 100 µL PBS) was administered intravenously into tumor-bearing mice and mice were continuously monitored for 24 h (Figure 5c). As expected, BSA-SMF800 showed preferential tumor uptake within 4 h post-injection, and the tumor fluorescence signals were apparently diminished at 24 h post-injection. Therefore, we confirmed the preferential tumor uptake of BSA during a certain period of time. This work suggests a simple and rational approach to utilizing various biomolecules for target-specific NIR fluorescence imaging after conjugation with the conjugatable small molecular SMF800 fluorophore.

## 4. Discussion

In the present study, a new type of conjugatable small molecular NIR fluorophore SMF800 was readily synthesized. Direct covalent conjugations of SMF800 to biomolecules demonstrated target-specific fluorescence imaging with low nonspecific background uptake. SMF800 exhibited appreciable optical properties and rapid body clearance behavior, which is principally important for practical issues of conventional NIR fluorophores, because nonspecific background fluorescence and low target site specificity were mostly created by the nonspecific uptake of the NIR fluorophores when using targeted bioimaging after conjugation with small-molecule drugs, polymers, and proteins [18,19]. In this regard, the low background uptake behavior of the SMF800 itself was consistently observed in all of the SMF800 conjugates prepared through the conjugation of SMF800 to the various molecular weight molecules from the small-molecule drug PAM (MW = 235 Da) to BSA (MW = 66,463 Da). This result demonstrated that SMF800 could be an ideal labeling agent for targeted NIR fluorescence imaging with higher fluorescence signal-to-background ratios compared to that of commercially available anionic NIR fluorophores such as IRDye800CW and Cy5.5 [19].

Furthermore, one of the important advantages of this study is the simple and efficient conjugation reaction using DMT-MM as a coupling agent in water for the SMF800 conjugates. In general, the activation of carboxyl groups with N-(3-dimethylaminopropyl)-N’-ethylcarbodiimide and N-hydroxysuccinimide (EDC/NHS) is the standard and well-established method for the formation of amide bonds from carboxylic acids and amines in aqueous solutions [31]. For this EDC/NHS chemistry, accurate pH control or buffers are necessarily required to be efficient. However, this pH shift during the reaction leads to a process of complexity and lower efficiency. Alternatively, the use of organic solvents generates a multistep process for purification. More importantly, EDC leads to the formation of toxic by-products such as N-acylurea, which should be removed after the coupling reaction [32]. Compared with the EDC/NHS chemistry, an efficient carboxylic acid activator DMT-MM is highly soluble and stable in water for several days at least [33]. The increased efficiency of DMT-MM over EDC/NHS can strongly contribute to the ultimate success of SMF800 conjugations with biomolecules. The main advantages of DMT-MM activation are: (i) no need for pH shift during the reaction (i.e., base-free reaction); (ii) no need for buffering the reaction mixture; (iii) improved yields at parity of feed ratio; (iv) no irritants and less toxic than carbodiimide-based reagent; (v) cost-effectiveness and capability of recycling [31,34]. Therefore, the SMF800 conjugates prepared in this study can also be diluted with 0.9% saline after the conjugation reaction and directly used for in vivo optical imaging without further purification.

To highlight the versatility of SMF800, the SMF800 conjugates were readily prepared by a simple and efficient one-pot synthesis approach and successfully used for targeted in vivo fluorescence imaging in real time. Based on the chemical structure of SMF800, the current work is the first report of the development of a small molecular NIR fluorophore consisting of a carboxylated indole moiety and a hydrophilic cinnamaldehyde moiety. To be considered with the ongoing issues of conventional NIR fluorophores, including complicated synthetic processes and persistent nonspecific background uptake, this simple and rational-design approach could suggest a clue to develop the contrast agents for potential future clinical applications.

## Data Availability

Data are contained within the article.
